# Discovery of 33mer in chromosome 21 – the largest alpha satellite higher order repeat unit among all human somatic chromosomes

**DOI:** 10.1038/s41598-019-49022-2

**Published:** 2019-09-02

**Authors:** Matko Glunčić, Ines Vlahović, Vladimir Paar

**Affiliations:** 1 Faculty of Science, University of Zagreb, 10000 Zagreb, Croatia; 2 Algebra University College, Ilica 242, 10000 Zagreb, Croatia; 3 Croatian Academy of Sciences and Arts, 10000 Zagreb, Croatia

**Keywords:** Computational models, Data processing, Genome assembly algorithms

## Abstract

The centromere is important for segregation of chromosomes during cell division in eukaryotes. Its destabilization results in chromosomal missegregation, aneuploidy, hallmarks of cancers and birth defects. In primate genomes centromeres contain tandem repeats of ~171 bp alpha satellite DNA, commonly organized into higher order repeats (HORs). In spite of crucial importance, satellites have been understudied because of gaps in sequencing - genomic “black holes”. Bioinformatical studies of genomic sequences open possibilities to revolutionize understanding of repetitive DNA datasets. Here, using robust (Global Repeat Map) algorithm we identified in hg38 sequence of human chromosome 21 complete ensemble of alpha satellite HORs with six long repeat units (≥20 mers), five of them novel. Novel 33mer HOR has the longest HOR unit identified so far among all somatic chromosomes and novel 23mer reverse HOR is distant far from the centromere. Also, we discovered that for hg38 assembly the 33mer sequences in chromosomes 21, 13, 14, and 22 are 100% identical but nearby gaps are present; that seems to require an additional more precise sequencing. Chromosome 21 is of significant interest for deciphering the molecular base of Down syndrome and of aneuploidies in general. Since the chromosome identifier probes are largely based on the detection of higher order alpha satellite repeats, distinctions between alpha satellite HORs in chromosomes 21 and 13 here identified might lead to a unique chromosome 21 probe in molecular cytogenetics, which would find utility in diagnostics. It is expected that its complete sequence analysis will have profound implications for understanding pathogenesis of diseases and development of new therapeutic approaches.

## Introduction

Tandemly repeated DNA sequences, known as satellites, form a substantial part of genome in many eukaryotes, including humans^1–3^. Some links between satellites and phenotypes have been established, for example, satellite depression was associated with cancer outcomes^4^, with chromosome missegregation and aneuploidy^2^, and with aging^5^. Satellite arrays form essential chromosome structure, such as centromeres and telomeres^3^, and they show astonishing variation in both sequence and copy number. However, in spite of their crucial importance, satellites have been understudied^6,7^.

The most abundant constituent of centromeres in human and other primate chromosomes are repetitive but rather divergent alpha satellite monomers of ~171 bp^8^. They are organized mostly as tandem repeats of *n*mer higher order repeat (HOR) copies, each consisting of *n* monomers, or as monomeric arrays without any HORs (Supplementary Fig. S1 and Table 1). Divergence among HOR copies within HOR array is about a few percent, while divergence among monomers within each HOR copy is sizably larger, ~20 to 40%^9,10^.

**Table 1:** Alpha satellite *n*mer HOR arrays (*n* ≥ 8) in hg38 sequence of human chromosome 21.

*n*	HOR copies	Complete HOR copies	HOR start position	Monomers in HOR	HOR array length (bp)	HOR unit length (bp)	HOR divergence (%)	Monomer divergence (%)
33	4	4	10,864,568	132	22,537	5639	5	19
23	23	18	10,887,205	517	88,022	3915	4	22
23*	20	11	7,970,290	440	74,877	3915	2	20
22	6	5	11,093,195	121	20,672	3758	3	17
20	16	15	10,975,327	316	54,133	3425	3	23
20	19	18	11,029,561	371	63,536	3425	4	21
16	16	2	11,124,080	465	22,561	2733	5	17
16	4	1	11,113,966	39	6,669	2736	4	17
11	712	12	12,283,230	3,722	632,586	1870	3	21
8	4	1	11,120,735	19	3,246	1368	6	17
8	854	826	11,146,741	6,650	1,134,213	1364	4	22

1^nd^ column: Number of monomers in HOR unit. Asterisk (*) denotes reverse monomer sequence in hg38 assembly with respect to the other HORs. 2^rd^ column: Number of HOR copies in the HOR array. 3^th^ column: Number of complete HOR copies in the HOR array. 4^th^ column: Start position of HOR array in genomic sequence of chromosome 21. 5^th^ column: Number of monomers in HOR array. 6^th^ column: Length of HOR array (bp). 7^th^ column: Length of HOR repeat unit (bp). 8^th^ column: Mean divergence among HOR copies (%). 9^th^ column: Mean divergence among monomers within HOR copies. Mean divergence is rounded off to nearest integer value. The divergence among HOR copies is determined as the mean value of divergence between pairs of the corresponding monomers in both HOR copies.

Several studies provided an evidence of functional role for satellite DNA^2,11–15^. They are implicated in centromeric functions, such as segregation in mitosis and meiosis, essential during cell division, pairing of homologous chromosomes, sister chromatid attachment and formation of kinetochore structures^11,12^. However, the interplay between genome sequences and the network involved in kinetochore ensemble is poorly understood^11,12,16–18^. While a number of centromeric proteins share homology among evolutionary distant organisms, one of challenging problems is that centromeric DNA sequences differ significantly even among closely related species and evolve rapidly during speciation^19^. Paradoxically, although the centromere’s role is conserved throughout eukaryotic evolution, the sequences that accomplish centromere function in different organisms are not conserved^16^. The functional importance of centromeric sequences notwithstanding, these regions of human genome remain poorly understood at the level of sequence ensemble and annotation^20^. Today, there are unique challenges of studying human satellite DNAs and RNAs and it is pointed toward technologies that will continue to advance our understanding of this, still largely untapped portion of the genome^21,22^.

In the past decade there have been impressive improvings in sequencing technology with the Next generation sequencing and Third generation sequencing/Long read methods including whole genome sequencing^7,23–26^. Unlike reads shorter than the underlying repeat structure, long reads allow direct inference of satellite higher order repeat (HOR) structure^27^.

Several computer algorithms for identification and analysis of tandem repeats in DNA sequences were previously designed and widely used, notably^28–32^. Two novel computational algorithms were recently designed and validated, convenient for HOR identification in novel centromeric repeat sequences obtained with long read sequencing^6^ GRM - Global Repeat Map^33–35^ and Alpha-CENTAURI^26,27^, convenient for HOR identification in novel centromeric repeat sequences obtained with long read sequencing^7^. The GRM algorithm is characterized by robustness with respect to deviations from regular repeat pattern, applicability to HORs with long repeat units and to long DNA sequences^33,34,36^. It is used in this study to identify alpha satellite HORs in hg38 genomic sequence of human chromosome 21. The advantage of using new human genome assembly hg38 (GCA_000001405.15) for alpha satellite HOR studies is that it adds a number of alpha satellite sequences to human chromosome 21^37,38^. A number of human chromosome 21p clones have been added to the new assembly and the centromeric gap was filled with “reference models”, which are representations of alpha satellite HOR domains.

## Results

**Figure 1:**
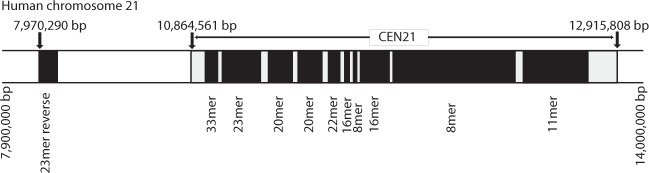
Alpha satellite HOR ideogram for linear positioning of alpha satellite HOR arrays with long repeat units (*n* ≥ 8) obtained by applying GRM algorithm to the hg38 assembly sequence of human chromosome 21. CEN21 denotes location of the centromere. Only a segment of chromosome 21 containing alpha satellite HOR arrays is displayed. Ten HOR arrays are located within the centromere. The 23mer HOR with reverse monomers in the long arm of chromosome 21 is removed far from the centromere. Closer description of HOR arrays is given in Table 1.

Here, the recent DNA sequence hg38 of human chromosome 21 is analyzed to identify and study computationally the complete ensemble of alpha satellite HORs embedded in DNA sequence, using robust GRM algorithm (Methods). Alpha satellite HOR ideogram obtained in this way for chromosome 21 is shown in Fig. 1. To our knowledge, this is the first time that a complete ensemble of *n* ≥ 8 alpha satellite HORs of a human chromosome was determined for the centromeric region.

**Figure 2:**
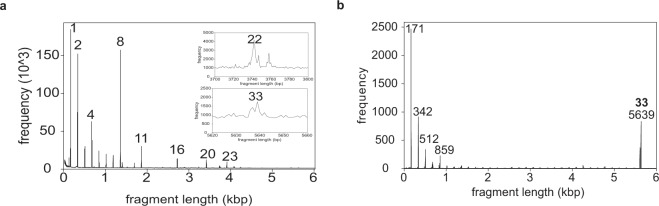
GRM diagrams for the whole human chromosome 21 and for the contig NT_187321 which contains 33mer HOR array. (**a**) GRM diagram for the whole chromosome 21. Pronounced peaks that correspond to alpha satellite HORs are denoted by number of monomers in *n*mer HOR repeat unit. Inserts give magnified presentation of weak peaks for 22mer and 33mer, which are sizably screened by a noise of different other repeats in the whole chromosome 21. (**b**) GRM diagram for contig NT_187321 in which the 33mer HOR array is located. The pronounced GRM peak at 5,639 bp is a signature of 33mer HOR (5,639: 171 ≈ 33).

The computed GRM diagram for hg38 DNA sequence of the whole chromosome 21 is shown in Fig. 2a. Pronounced peaks at fragment lengths that are approximately equal to 171·*n* bp, i.e., to multiples of alpha satellite monomer length 171 bp, are candidates for *n*mer alpha satellite HORs, especially if a peak at ~171·*n* bp is sizably higher than the neighboring peak at lower fragment length ~171·(*n-1*) bp. For example, for 8mer HOR, the peak at ~171·8 bp is sizably stronger than the peak at ~171·7 bp; for 11mer HOR, the peak at ~171·11 bp is sizably stronger than at ~171·10 bp; for 16mer HOR, the peak at ~171·16 bp is sizably stronger than at ~171·15 bp; for 20mer HOR the peak at ~171·20 bp is sizably stronger than at ~171·19 bp, etc. We can directly confirm this attribution by analyzing the corresponding DNA sequences. For monomeric alpha satellite arrays the frequencies of peaks at 171·*n* bp gradually decrease with increasing *n* and a peak sizably above this background is an indication for HOR.

In order to identify complete ensemble of alpha satellite HORs in a given DNA sequence we extended GRM algorithm with introduction of a novel algorithm ALPHAsub (Methods) to identify positions of all alpha satellite arrays (regardless weather of HOR type or nonHOR type) in DNA sequence. In this way we determine contigs in which alpha satellite arrays are located, i.e., to each alpha satellite array the corresponding contig is assigned. Then we apply GRM to each of these contigs. The GRM analysis of contigs containing alpha satellite *n*mer HOR provides a sizably more pronounced peak at position 171·*n* bp, than the GRM analysis of the whole genome, because the noise due to other repeats is sizably smaller for a contig than for the whole chromosome. In this way it is straightforward to determine whether the alpha satellite array is HOR.

The GRM peak corresponding to 33mer in GRM diagram for the whole chromosome 21 is small, but visible at 5639 bp in the magnified segment of HOR diagram. On the other hand, the 5639 bp peak of 33mer HOR is sizeable in GRM diagram for contigs NT_187321.1, in which the 33mer HOR is, located (Fig. 2b). The length of a 33mer HOR copy is ~33 × 0.171 kb ~5.6 kb.

**Figure 3:**
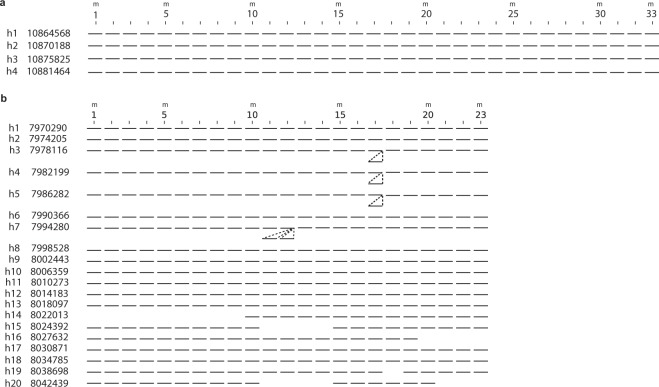
Schematic presentation of aligned monomer structure of 33mer HOR and 23mer HOR (reverse) arrays in human chromosome 21. (**a**) 33mer (4 complete HOR copies). Top: enumeration of columns corresponding to 33 constituent consensus monomers (Nos. 1 to 33, enumeration of every fifth monomer is displayed). Each of the four 33mer HOR copies (denoted *h*_*i*_, *i* = 1, 2, 3, 4) is presented by 33 bars in the *i*th row. Each monomer of the same type (from consensus HOR) in different HOR copies is presented by a bar in the same column, corresponding to monomer enumeration at the top. (**b**) 23mer with reverse monomers (20 HOR copies). Each of the twenty 23mer HOR copies (denoted *h*_*i*_, *i* = 1, 2, … 20) is presented by 23 bars in the *i*th row.

Schematic presentation of aligned monomer structure of 33mer HOR array is presented in Fig. 3a. This HOR array has very regular structure; it’s all four HOR copies are complete. Using GRM, the DNA sequences of 33mer HOR copies are determined from hg38 for human chromosome 21 and the consensus sequence was determined (Supplementary Table S1). The average divergence among monomers within the 33mer consensus HOR is 19%, and divergence between 33mer HOR copies in HOR array is 5%. The 23mer HOR starting at the chromosome position 7,968,750, far from the centromeric region, has reversed monomers with respect to the other ten HORs. This 23mer HOR array consists of 20 HOR copies. Schematic presentation of aligned monomer structure of 23mer HOR (reverse) array is presented in Fig. 3b.

**Figure 4:**
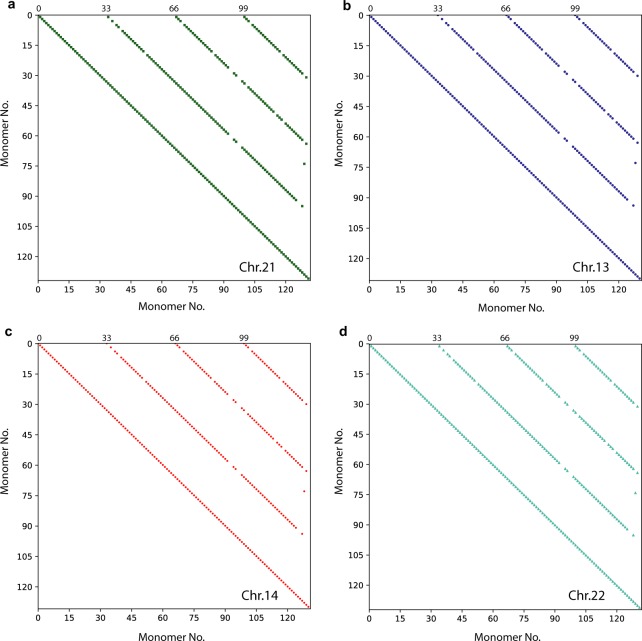
Dot-matrix plots of 33mer HORs in four acrocentric chromosomes. (**a**) chromosome 21; (**b**) chromosome 13; (**c**) chromosome 14; (**d**) chromosome 22. Dot-matrix analyses determined the presence or absence of HOR structure using a window size of monomer length and mismatch limits ranging at ~7%. Monomers are labeled in order of appearance, displayed in matrix along the upper horizontal axis (from left to right) and along the left vertical axis (from up to down): at both axes label 1 corresponds to the first monomer in alpha satellite ensemble, label 2 to the second monomer, etc. In this way, the alpha satellite ensemble is compared with itself, giving pairwise comparisons of divergence between constituting alpha satellite monomers. Each cell in dot-matrix which represents divergence between monomers located at identical positions in different HOR copies (e.g. the second monomer in the third HOR copy on the horizontal axis and the second monomer in the fourth HOR copy on the vertical axis, etc.) correspond to relatively small divergence between monomers (here chosen below 7%) and is shown as colored dot. The other cells in dot-matrix correspond to higher divergence (above 7%) are blank. In this way, for each HOR array the dot-matrix diagram is obtained as a set of equidistant diagonal lines at spacing equal to the number of monomers in HOR unit (*n* = 33), parallel to the self-diagonal.

Additionally, for each identified alpha satellite array we computed the corresponding dot-matrix diagrams to determine whether it has a HOR structure. The dot-matrix for 33mer HOR in human chromosome 21 is shown in Fig. 4a. The HOR pattern is characterized by diagonal lines at the spacing of *n* = 33 monomers, parallel to the self-diagonal. We computed dot-matrix diagrams for all high-multiple HORs in chromosome 21. For example, dot-matrix diagrams are shown for 23mer, 23mer (reverse) and 22mer HOR arrays containing 517, 448 and 371 monomers, respectively (Supplementary Fig. S4).

In accordance with GRM diagrams (Fig. 2a and Supplementary Fig. S3a–c) and dot-matrix analysis (Fig. 4a–d), we find for hg38 assembly the 100% identical 33mer HORs in chromosomes 21, 13, 14 and 22, and also there are gaps in hg38 in the neighborhood of 33mers. A more complete sequencing of this region seems to be required.

## Discussion

DNA sequence of the whole human chromosome 21 was previously used in studies of gene catalogue^39^. The earlier restriction map estimates^40,41^ for HORs with long repeat units largely differ from the present GRM results in Fig. 1. For HOR repeat units longer than 20mers we identified here two 20mers, one 22mer, two 23mers and one 33mer, while earlier restriction map estimates in this range indicated one 22mer and one 28mer^40,41^. For *n*mers below *n* = 20 monomers, the previously predicted 11mer and 16mer^40,41^ are in accordance with present results, but for other *n*mers previous estimates show some differences.

Extensive earlier work on alpha satellite sequences and satellite DNA in centromere near regions of chromosome 21 was summarized in monograph^42^. Recent study of alphoid sequences revealed five distinct alphoid clusters Mp1-Mp5 that are large in size (25–189 kb each) and extend over a distance at least 5 Mb from D2171.

Using a different method, alpha satellite arrays in hg38 sequence of chromosome 21 were recently studied^37^ and their results for positions of alpha satellite arrays in centromere were obtained which correspond to our GRM results, but there were also some pronounced differences. In ref.^37^ only four alpha satellite HORs have been identified out of eleven identified here using GRM and, among others, the 33mer HOR was not identified. Also, the pronounced reverse alpha satellite array far outside of the centromeric region which was identified here using GRM, was not found in ref.^37^.

Three of our eleven HOR arrays, 11mer, one of two 23mers and 22mer, can have attributions as follows. The 11mer HOR corresponds to the well known D21Z1 sequence, one of our 23mer HORs to D21Z3 (pTRA-1) with 3.9-kb repeat unit positioned in Mp3 cluster, and our 22mer to SF5 HOR of ~3.7 kb (GJ212128), a part of which is almost identical to the pTRA-7 probe corresponding to D21Z7.

The 33mer HOR identified here for chromosome 21 is the longest alpha satellite HOR repeat unit reported so far among all 22 somatic chromosomes. Evidence for a slightly longer HOR repeat unit, a 35mer, was found previously only in the sexual chromosome Y^43^.

Previous analysis of hybridization of alpha satellite DNA showed that chromosomes 13 and 21 share a 4mer HOR array and that the HOR unit is indistinguishable on each chromosome, while the 6mer HOR array is shared by chromosomes 22 and 13, distinguishable from the 13/21 4mer HOR array^44^. These results suggested that, at some point after they originated and were homogenized, different subfamilies of alphoid sequences must have exchanged between chromosomes 13 and 21 and separately between chromosomes 13 and 22. Following homogenization of one chromosome, a portion of HOR array could have been transferred between nonhomologous chromosomes by recombination. If this recombination event had happened recently in evolutionary time, any independent homogenization process operating on the two blocks of alpha satellite DNA, now separated on different chromosomes, may not have had sufficient time to cause isolated sequences to diverge^44^.

Chromosome identifier probes are largely based on detection of higher order alpha satellite repeats and it has been a constant thorn in the side of molecular cytogeneticists that the probe for chromosome 21 also picked up chromosome 13 (and vice versa). In this respect one could comment on the likelihood of recent work leading to a unique chromosome 21 probe, which would find great utility in diagnostics. To this end, in Table 2 we present the alpha satellite *n*mer HOR arrays (*n* ≥ 8) identified here in hg38 sequence of human chromosome 13 for comparison with Table 1 for chromosome 21. Here we find some pronounced differences between alpha satellite HORs in chromosomes 21 and 13. In chromosome 21 we identify four complete 33mer HOR copies and in chromosome 13 three (in one HOR copy two monomers are deleted). A significant difference exists for 23mer HORs: we identify two distinct 23mer HOR arrays in human chromosome 21 (constituted of 23 and 20 HOR copies, respectively), while in chromosome 13 we identify only one HOR array (constituted of 23 HOR copies). This provides a pronounced distinction between chromosomes 21 and 13. We also note a sizable difference between 16mer HORs: we identify two distinct 16mer HOR arrays in human chromosome 21 (constituted of 16 and 4 HOR copies, respectively), while in chromosome 13 we identify only one HOR array (constituted of 3 HOR copies). Finally, we note a difference between lengths of long 8mer HOR arrays in chromosomes 21 and 13 (854 and 832 HOR copies, respectively).

**Table 2:** Alpha satellite *n*mer HOR arrays (*n* ≥ 8) in hg38 sequence of human chromosome 13.

*n*	HOR copies	Complete HOR copies	HOR start position	Monomers in HOR	HOR array length (bp)	HOR unit length (bp)	HOR divergence (%)	Monomer divergence (%)
33	4	3	16,000,179	131	22,366	5639	5	20
23	23	15	16,022,645	515	88,022	3915	4	21
22	6	5	*16*,*228*,*806*	120	20,672	3758	3	17
20	16	15	*16*,*110*,*767*	316	54,133	3426	3	24
20	19	18	*16*,*165*,*001*	371	63,536	3425	4	22
16	3	2	*16*,*249*,*406*	39	6,670	2736	5	17
11	363	229	*17*,*418*,*670*	3,721	632,415	1871	3	23
8	3	2	*16*,*256*,*175*	20	3246	1368	6	17
8	832	818	*16*,*282*,*181*	6,646	1,134,112	1365	4	23

1^nd^ column: Number of monomers in HOR unit. 2^rd^ column: Number of HOR copies in the HOR array. 3^th^ column: Number of complete HOR copies in the HOR array. 4^th^ column: Start position of HOR array in genomic sequence of chromosome 13. 5^th^ column: Number of monomers in HOR array. 6^th^ column: Length of HOR array (bp). 7^th^ column: Length of HOR repeat unit (bp). 8^th^ column: Mean divergence among HOR copies (%). 9^th^ column: Mean divergence among monomers within HOR copies. Mean divergence is rounded off to nearest integer value. The divergence among HOR copies is determined as the mean value of divergence between pairs of the corresponding monomers in both HOR copies.

Analogously, it could be hypothesized here that at some point after it originated and was homogenized on one of the chromosomes 21, 13, 14 or 22, the 33mer HOR could have been transferred recently in evolutionary time by recombination to the other chromosomes.

Recently, almost complete genomic sequences open the possibility to determine complete ensemble of alpha satellite HORs in the whole human genome, which in turn enables a broad investigations of alpha satellite HORs and monomeric repeats and their influence on centromere dynamics. As noted by van Dijk *et al*.^25^, ultralong reads may allow complete, gapless assembly of human genomes in the near future, which will further boost human genetic research and personalized medicine^25^. Among others, complete DNA sequences will open new challenges related to HORs as possibly important regulatory elements. One could hypothesize that the richness of different long HOR repeat units will be found in centromere of other chromosomes too. We are only at the beginning of the third revolution in sequencing technology and the coming years may bring exciting new developments in HOR studies.

## Methods

### Sequence data

In this study the hg38 assembly sequence of chromosome 21 was used for HOR analysis.

### ALPHAsub algorithm

The novel method of identifying alpha satellite arrays in DNA sequence is as follows. As “ideal key word”, we use a robust 28-bp segment from alpha satellite DNA sequences, TGAGAAACTGCTTTGTGATGTGTGCATT and its reverse complement. First, using the Levenshtein distance algorithm, all positions in the whole chromosome are determined where the 28-bp sequence of “ideal key word” or its reverse complement differs from a “real key word” by at most nine nucleotides. Second, the distances between positions of neighboring “real key words” are calculated. Third, only those “real key words” are retained for which distance to its previous neighbor is approximately equal to 171 bp or to a multiple of 171 bp (d(*n*, *n-1*) ~ *m*·171; *m* = 1, 2,…). In the latter case (*m* > 1), the additional “real key words” (one for *m* = 2, two for *m* = 3, and so on) are determined in the sequence between “real key word” and its previous neighbor, using the Levenshtein distance algorithm, at positions with the smallest difference of “real key words” compared to “ideal key word” or its reverse complement. In general, a distance between the additional “real key words”, obtained by this method, is always approximately equal to 171 bp. In this way, we determined positions of all alpha satellites within chromosome 21. In the next step, using positions of “real key words”, all alpha satellites from chromosome 21 hg38 DNA sequence are extracted and different alpha satellite ensembles are identified. On this basis, we have designed our ALPHAsub algorithm and computer program. Applying ALPHAsub program to the hg38 sequence of chromosome 21 we determine location of all alpha satellite arrays within genomic sequence.

### GRM algorithm

Global repeat algorithm (GRM) is an efficient and robust novel method to identify and study repeats, especially HORs, in a given DNA sequence^33–35^. To identify alpha satellite HORs, we compute GRM diagrams for genomic sequence of the whole chromosome. For long DNA sequences of whole chromosomes, due to many repeats in genomic sequence the noise in GRM diagram increases with increasing length of HOR repeat unit. This noise is significantly reduced by applying GRM to those regions which contain alpha satellite arrays. Such regions are first selected using ALPHAsub algorithm for analysis of the whole chromosome sequence. The novelty of GRM approach is a direct mapping of symbolic DNA sequence into frequency domain using complete *K*-string ensemble instead of statistically adjusted individual *K*-strings optimized locally. In this way, GRM provides a straightforward identification of DNA repeats using frequency domain, but avoiding mapping of symbolic DNA sequence into numerical sequence, and uses *K*-string matching, but avoiding statistical methods and locally optimizing individual *K*-strings. For a given sequence, the GRM algorithm provides in the first step (“identification step”) the corresponding GRM diagram; each significant peak (“fragment length”) presents the length of repeat unit. In the second step (“analysis step”), for each significant GRM peak the algorithm determines corresponding repeat sequences and their positions, the consensus repeat unit and divergence between repeat copies and with respect to consensus.

### GRM algorithm expanded by ALPHAsub algorithm

Successive application of ALPHAsub and GRM algorithms is used for identification and analysis of alpha satellite HORs in a whole chromosome sequence: in the first step we identify contigs that contain alpha satellite arrays and in the second step we perform GRM computation for these contigs. In this way, an ensemble of all alpha satellite HORs is extracted from a given genomic sequence (here hg38).

### ALPHAsub algorithm expanded by Dot-matrix method

For each alpha satellite array identified by ALPHAsub algorithm, the corresponding dot-matrix diagrams are created to identify alpha satellite HORs on the basis of equidistant lines parallel to self-diagonal.

## Supplementary information

Supplementary Tables and Figures

## Data Availability

Further code information is available on request from the authors.
