# Supplementary material for: Discovery of 33mer in chromosome 21 – the largest alpha satellite higher order repeat unit among all human somatic chromosomes
Source: Sci Rep. 2019 Sep 2;9:12629. doi: 10.1038/s41598-019-49022-2 (PMC6718397; doi:10.1038/s41598-019-49022-2)

Discovery of 33mer in chromosome 21 – the largest alpha satellite higher order repeat unit among all human somatic chromosomes

Matko Glunčić[1], Ines Vlahović[1], Vladimir Paar[1,2]

[1]Faculty of Science, University of Zagreb, 10000 Zagreb, Croatia. [2]Croatian Academy of Sciences and Arts, 10000 Zagreb, Croatia.

Supplementary Tables and Figures

TGAAAAACATCTTTGTGATGTGTGCATTCATCTCAAAGAGTTCAACCTTTCTTTTGATTGAGCACTTTTGAAATACTTTTTGGAGAATC
TGTAAGTGGATATTTGGAGGGCTTTGGGTCCTATGGTGGTAAAGGAAACATCTTCACATAAAAACTACACAGAAGCATTCTGAAATACC
TCTTTGTGATGCTTGCATTCATCTCACATAGTTGAACCATTCTTTTTATTGAGCAGTTTTGAAACAATCTCCTTGTAGAATGTGCAAGT
GGATATTTGGAACGCTTTGATGAGTATGGTGGAAAATGAAAAATCTTCACATAAAAACTAGACAGAAGTACTCTGAGAAACTTCTTTGT
GATGTGCACATTCATCTCACAAATTTGAAAATTTCTTTTGATTGAGCAGTTTTGAAACGCTCTTTTTCTAGAATCTGCCAGTGGATATT
TGGAGTGCTTTGAGTCCTATGGTGGAGAAGGAAATATCCTCACATAAAAACTAGAGAGAAGCATTCTGAGAAACTTCTTTGTGATGTGT
GCATACATCTCACAGAGTTGAAACTTTCTATTGATTTAGCATTTTTTATACACTTTTTGTAGGATCTGCAGTTGCTATTTGGAGCCCTT
TGGGGCCAATGGTGGAAAAGTATTATCTTCTCATAAAAACTAGACAGAAGCATTTTGAGAAACTTCTCTGTGATGTGTTCATTCATCTC
ACAGATTTGAACCATTCTTTTGATTCAGCAGTTTTGAAACACTCTTCGTAGAATCTGCAAGTGCATATTTAGATCGCTTTGAGAAGTGT
GGTGGAAAAGGAAATATCTTCACATAAACACTAGACAGAAGCATTCTGAGAAACGTCTTTGTGATGTGTCCATTCATCTCACAGAGTTG
AAACTTTCTTTTCATTGAGCAGTTTTGAAACACTCTTTTTATAGAATCTGCAAGTGGATATTTGGAGCGCTTTGGAGAGAATGGTGGAA
ATGGAAATATCTTCATATAAAAACTACGGAGAAGCATTCTGAGAAACGGCATTGTTATGTGTGCCTTCAGCTCACAGAGTTGAACCTTT
CTTTTGATTGAGCAGTTTTGAATCCCTCTTTTTGTAGAATCTGCAAGTGGATATTTGGAGAGCTTTAGGGCCTATGGTGGAAAAGGAAA
TATCTTCACATAAAAACTACACAAAAGCATTCTGAGAAACTTCTTTCTGATGTGTGCATACAACTCCCAGAGTTGAATCTTTCTTTTGA
TTGTGCAATTTTGAAACACTTCTTTTGTAGAATCTGCAAGTGGATATTCGGAGGGCTTTGCCGAGTATAGTGGAAAAGGAAATAACTTT
GGATAAAAGGTAGACAGAAACATTCTGAGAAACTTCTTTGTGATGTGTGCATTCAACGTACAGAGTTGAACCTTTCTTTAGATCGGGCA
GTTTTGAAACACTATTTTTGTAATATCTGCAAGTGGATATTTGGTGACCATTGCAGCCTATGGTGGAAAGGCAAATATCTTCACATAAA
AACTAGACAGAAGCATTCTGAGAAACTTCTTTGTGATGTGTGCATTCATCTCACACAGTTCAACTTTTCTTTTGATTCAGCAGTTTGGA
AACAGTATTTTTCTACAATCTGCAAAGGGATACTTCTTAGCCGATTTAGGCCTATGGTGAATTAGGAAATATCTTCACATAAAAAATAA
ACAGAAGCTTTCTGAGAAACTTCTTTGGGATGTGTGTTTTCATCTCACAGAGATGAAACTTTCTTTTGATTGAGCAATTTCGAAACTCT
CTTTTTGTAGGATCTGCAAATGGATATTTGGAGCGCTTTGAGGCCTGTGGTGAAAAAGGAAATATCTTCACATAACAACCAGACAGAAG
CATTCTGGAAACATCTTTGTGATGTGTGCATTCATCTCACAGAGTTGAACCTTTCTTTTGATTGAGCAGTTTGGAAACAGTCTTTGATA
GTATCTGCAGAGAGATATTTGTGAGCATTTTGAGGACTTTGGTGAGAAAGAAAAAACCTCAAAAAAAAACCAAGCAAAAAGATTCTGAG
AAACTTCTTTGTGATGTGTGCATTCAACTGACAGAGTTGAAACTTTGTTTTGATTGAGCAGTTTGTAAACAGTCCTTTTGTAGGATCTG
CAAAGGGATAGTTCTGGGCCCATTGAGACCTATGGTGAAAGAAGAAATATCTTCACTTAAAAACTAGACAGAAGCATTCTGAGAAACTT
CTTAGTGATGTGTGCTTTCATCTCACAGGTTTGAACTTTCTTTTGATTGAGCAGTTTGGAAACAGTGTTTTTGTAGAATCTGCAAAGGA
TATTTTGAGCGCTTTGACGCCTATGGTGAAAAAGGACATATCTTCACATGAAATCTAAACAGAAGCTTTCTGAGAAACTTCTTTTTTAT
GAGTTCATACATCTCACAGAGGTGAAACTTTCTTTTCATTGAGCAGTTTGGAAACAGTCTTTTTGTACAGTCTGCAAAGGAAATTTCTG
CGAAGTTGGAGGCCTATGGTGAAAAAGAAATATCTTCAGATAAAATGTAGACAGAAGTATTCTGAGAAAATTTTTTGTGATGTATCTAT
TCATCTCACAGAGTTGAATTTTTCTTTTGATGGAGCAGTCTGGAAACAGTCTTTTTGTAGTATCTGAAGAGGGATATGTGAGAGCAGTT
TAAGGCCTGTGGTGAAAAAGGAAATATCTTCACATAAAAACAAGGTAGAAGCATTCTAAGAAACTTCTTTGTATTGTGTGCATTCATCT
CAAAGACTTGAACCTGTCTTTGGACTGAGCAGTTTGGAAACTGTCGTTTTGTAGAATCTGTGAAGGGATATTTCTGAGCCCATTGAGGC
CTATGGATGAAATAGGAAATATCTTCACATAAAAACTAGACAGAGGATTTCTGAGAAACTTCTTTGTGATATGTGGTTTCATCTCACAG
AGTTGAACCATTCTTTTGGTTGAGCAGTTAGGAAACAGTATTTTTGTAGGATCTGCAAAGGGATATTTCTGTTCCCATTGACGCCTATG
GTGAAAAAGGACATATCTTCACATAAAAACTAGACAGAAGCTTTCTGATAAACTTCTTAGTGATGTGTGCTTTCATGTCACAGATTTGA
AACTTTCTTTTGATTGAGCAGTTTGGAAACAGTCTTTTTGTAGAATCTGCAAATGGATATTTGGAGTGCTTTGAGGCCTATGGTGAAAA
AGGAAATACCTTCACATGAAATATAAACAGAAGCTTTCTGAGAAACTTCTTTTTGATGCATGCATACATCTCACAGAGTTGAAAGTTTC
TTTTCATTGAGCAGTTTGGAAACAGTCTTTTTGTACAATCTGGAATGGGATATTTCTGAGAAGTTGGAGGCCTATATCGAAAAAGAAAT
ATCTTCACATAAAAACTAGACAGAAGTATTCTGAGAAACTTCTTTGAGATGTATCCTTTCATCTCACAGAGTTGAACCTTACTTTTGAT
GGAGCAGTTTGGAGACAGTCTTTTTGTAGTATCTGCAGAGGGATATCTGAGAGCAGTTTAAGGCCTATGGTGAAAAAGGAAATATCTTC
ACATAAAAACTAGGCAGAAGCATTCTGAGAAACTTCTTTGTGATGTATGCATTCAACTCAAAGAGGTGAAACTTTCTTTGGATTGAGCA
GTTTGGAAACAGTCCTTTTGCAGAATCTGCAAAGGGATATTTCTCAGTCCATTGAGGCCTATGGTGAAATAGGAAATAACTTCCCATAA
AAACCAGACAGAAGGTTTCTGAGAAACTTCTTTGAGATATGTGCTTTCATCTCACAGAGCTGAACCTTTCTTTTGGCTCAGAAGTTTGG
AAACAGTCTTTGTGTAGAATCTGCAAAGCGCTATTTTTGAGCACCTTCTGGACTATGGTGAAACAGAAAATATCTTCACATAAAAACTA
GACAGAAGCTTTCTGAGAAACTTCTTTATGATGTGTTCTTTCATCTCACAGAGTTGTAACTTTCCTTTGATTGAGCAGTTTGGAAACAC
TCTTTATGGGGAATCTGCAAGTGGATATTTGGAGTCCTTTGTGGCCTATAGTGGAAAACGAAATATCTTCACATAAAAACTAGACAGAA
TCATTCTGAGAAACTTCTTTGTGATGTGCACATTCATCACAAAGAGTTGAACATTTCTTTCGATTCAGCAGTTTGGAAACAGTCCTTTT
GTAGAATCTGTGAAGGGATATTTCTCAGCCCATTGATGCCTATGGATGAAATAGGAAATATTCTCACATAAAAACTAGACAGAAAATTT
CTGAGAAACTTCTTTATGATATGTGGTTTCATCTCACAGAGTTGAACCGTTCTTTTGGTTGAGCAGTTTGGAAACACATTTTTTGTAGA
ATCTGCAAGTGGATATTTGGAGCACATTGAGGCCTATGGTGGAAAACGGAATATTTTCACATAAAAATTAGACAGAAGCATTCTGAGAA
ACTACTTTGTGATGTGTGCATTCAACCCACAGAGTTCAACCTTTCTTTTGATTCAGCAGTTTTGAAACACTCTTTTTGTAAAATCTGAC
AGTGGATTTTTGGAGCGCTTTGAGGCCTACGGTGGAAAAGGAAATATCTTCACATAAATAGTACACAGAAGCATTCTGAGAAACTTCTT
TGTGATGTGTGCATTTAACTCAAAGAGTGCAATCCTTCTTTAGATTGAGCAGTTTTGAAAGACTCCTTTTGCAGAATCTGCAAGTGGAT
GTTTGGAGCGCTATGTGGCCTTAAGTGGAAAAGGCAATATCTTCACATAAAAACTAGACAACAGCATTCTGAGAAACTTCTTTGTCATG
TGTGCATTCATCTCACAGAGTTGAAGCTTTCTTTTGATTGAGCAGTTTTGAAACACTCTTTTTGTAGAATCTCCAATTGGATACTTGGA
GCGTTTTGAGGCCTATGGTAGAAAAGTAAATATCTTCACGTGAAAACTACACAGAAGCATTCTGAGAAATTGGTTTGTGATGTGTGCAT
TCAACACACAGAGTTGAACCTTTCTTTTGATTGAGCAGTTTTGAAACACACTTTTTTTAGGATCTGCAAGTGGATATTTGGAGTGCTTT
GTGGCCTAATGCGGAAAAGGATATATTTTCACATAAAAACTACGGAGAAGCATTCTGAGAAACTTCTTTGTGATGTGTGCATTCATCTC
ACAGAGTTCAACCTTTCTTTTGATTGAGCAGTTTTGAAACACTCTTTTTGTAGAGTGTGCAAGCGGATATTTGGAGCTCTTTGAGGCTT
ATGGTGGAAAAGGAAATATCTTCACATAAAAACTACAGAGAAGCATTCTGACAAAGTTCTTTGTGTTGTGTGTGTTCAACTCACAGAGT
TGAGTCTTTCTTTTGATTGAGCAGTTTTGAAACACTCTTTTTTTAGAATCTGCAAGTGGATATTTCGAGTGCTTTGCAGCCTCTGTTGG
AAAAGGAAATATCTTCACATAAACTAGACAGAAGCATTCTGAGAAACTTCCTTGTGATGTGTGCATTCATCTCACAGAGTTGAAACTTT
CTTTTGATTGTGAAGTTTTCAAACACTCTTTTTGTAAAATCTGCAAGTGGATATTTGGAGGCCTTTGTGGCCTACAGGGGAAAAAGAAA
TATCTTCACATAAAAACTACACAGAAGCATTC

Supplementary Table S1 | Consensus sequence of 33mer HOR repeat unit in human chromosome 21.

Supplementary Figure S1 | Illustrative alignment scheme of monomeric and HOR arrays. a, Monomeric array of twelve monomers denoted *m1*, *m2*, ... *m12*. Pairwise sequence divergence among all monomers in the array is sizable, ~ 20 to 40 %. In the alignment scheme there are no two monomers of the same type and therefore all are displayed in the same line. b, HOR array of four 3mer HOR copies denoted *h1*, *h2*, *h3* and *h4*. Pairwise divergence among HOR copies is small. Near identity of HOR copies is a consequence of similarity (at identity level of more than ≈ 95%) among the corresponding monomers from all HOR copies: the first monomer in *h2*, *h3* and *h4* are very similar to the first monomer *m1* in *h1*, therefore they are all denoted *m1*; analogously, the second monomer in *h2*, *h3* and *h4* are very similar to the second monomer *m2* in *h1*, therefore they are all denoted *m2*; and the third monomer in *h2*, *h3* and *h4* are very similar to the third monomer *m3* in *h1*, therefore they are all denoted *m3*. Thus, each 3mer HOR copy approximately consists of three alpha satellite monomers, denoted *m1*, *m2*, *m3*. Divergence among four HOR copies: div(*hi*, *hj*) up to ~ 5%, (i, j = 1, 2, 3, 4; $i \neq j$). Divergence among monomers within each HOR copy: div(*mk*, *ml*) ~ 20 to 40% (k, l = 1, 2, 3 and $k \neq l$).

Thus, the HOR array is approximately

m1 m2 m3 m4 m5 m6 m7 m8 m9, m10 m11 m12 where

m1 ≈ m4 ≈ m7 ≈ m10 ≠ m2 ≈ m5 ≈ m8 ≈ m11 ≠ m3 ≈ m6 ≈ m9 ≈ m12.

In approximate monomer alignment scheme this can be presented as:

h1	*m1*	*m2*	*m3*
h2	*m4*	*m5*	*m6*
h3	*m7*	*m8*	*m9*
h4	*m10*	*m11*	*m12.*

Each column of monomers in that scheme corresponds to one monomer type (column 1 – monomers of type *m1*; column 2 – monomers of type *m2*; column 3 – monomers of type *m3*.) Each row corresponds to one HOR copy: (row 1 – HOR copy *h1*; row 2 – HOR copy *h2*; row 3 – HOR copy *h3*; row 4 – HOR copy *h4*). This pattern can be extended to *n*mers for any number of monomers *n*.

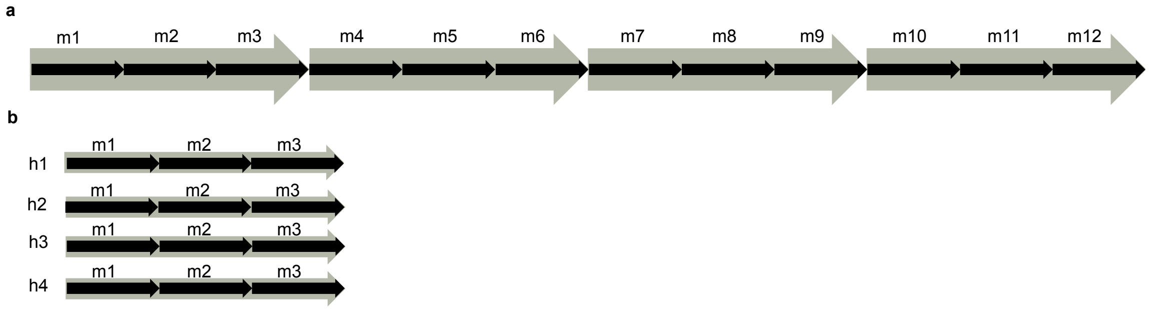

Supplementary Fig. S2 Dot-matrix diagrams for two 23mers and one 22mer HOR arrays in human chromosome 21. a, 23mer; b, 23mer (reverse); c, 22mer.

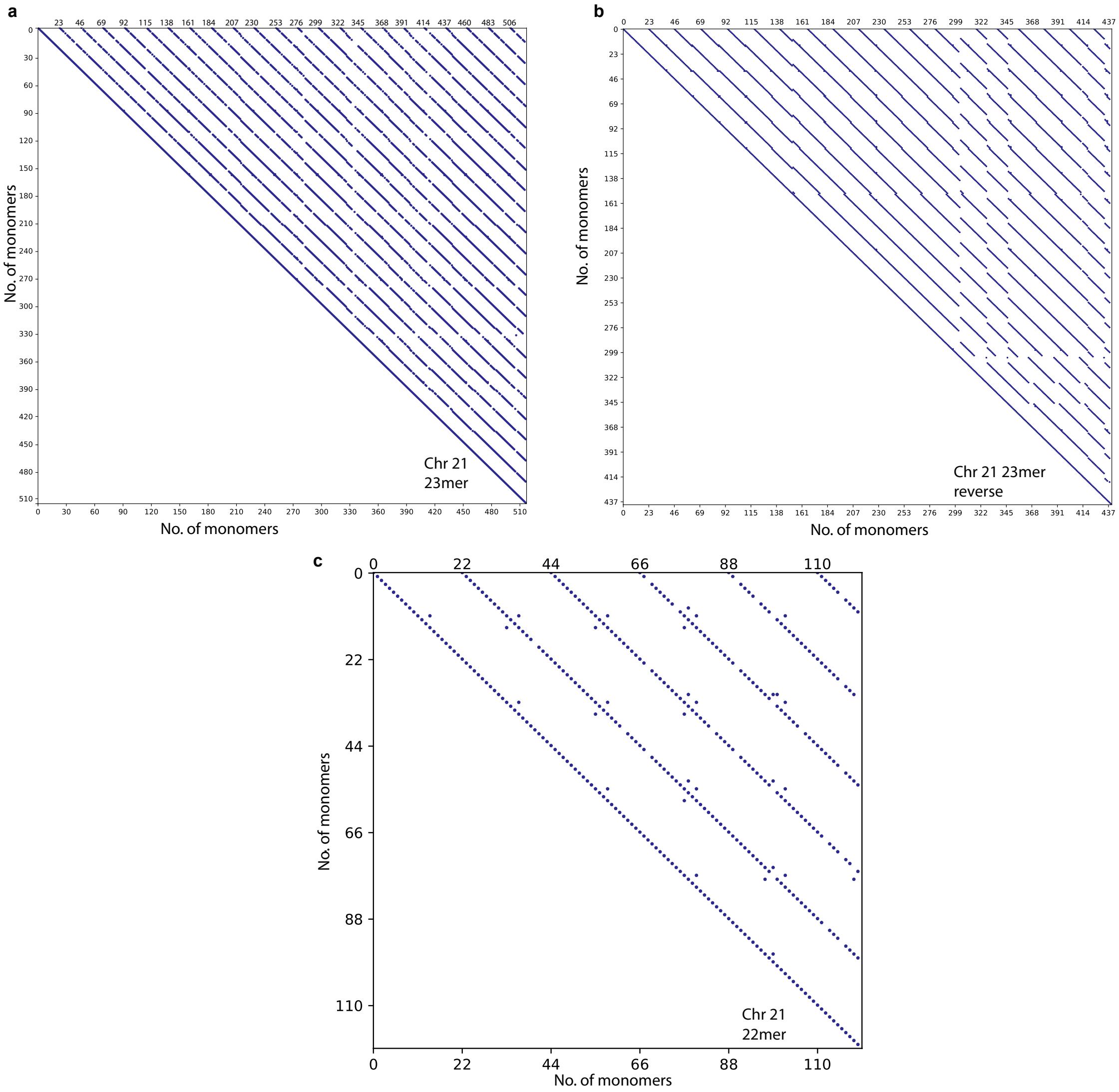

Supplementary Figure S3 | GRM diagrams for whole chromosomes 13, 14 and 22.
a, chromosome 13; b, chromosome 14; c, chromosome 22.

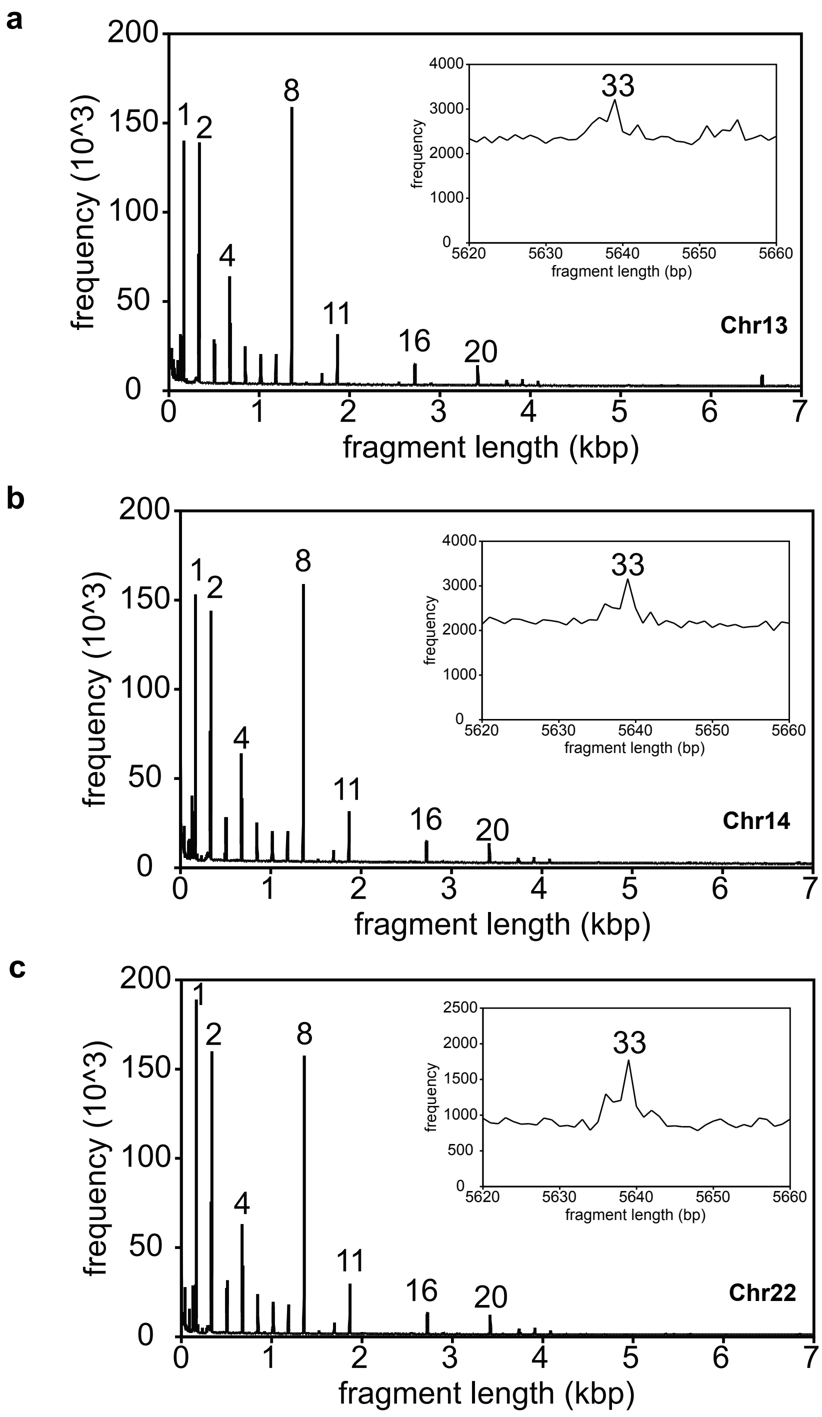

Supplement: Supplementary file 1 — Supplementary Tables and Figures [file 41598_2019_49022_MOESM1_ESM.pdf]
